# Lead exposure increases the risk of retinal vein occlusion: a population-based analysis and investigation of PRICKLE4/PLCXD1-mediated endothelial cell mechanisms

**DOI:** 10.3389/fpubh.2026.1826278

**Published:** 2026-05-12

**Authors:** Shuai Yang, Lvjie Xu, Tianxing Chen, Tengteng Yao, Xifang Zhang, Jiawei Wang, Zhaoyang Wang

**Affiliations:** 1 Beijing Tongren Eye Center, Beijing Tongren Hospital, Capital Medical University, Beijing, China; 2 Department of Pharmacy, Beijing Tongren Hospital, Capital Medical University, Beijing, China

**Keywords:** lead, phosphodiesterase, PLCXD1, PRICKLE4, retinal vein occlusion, Wnt signaling pathway

## Abstract

**Background:**

Retinal vein occlusion (RVO) is one of a leading cause of blindness with complex etiology. This study aims to investigate the relationship between blood heavy metal concentrations and RVO, and explored transcriptomic effects of lead on endothelial cells.

**Methods:**

Using the 2005-2008 NHANES data, individuals ≥40 years with fundus and heavy metal testing were analyzed. Multivariate logistic regression model was used to evaluate the relationship between blood lead, cadmium, mercury and RVO, with multi‑exposure modeling for combined effects. RNA sequencing was performed on HUVECs treated with 10 μM or 30 μM lead nitrate versus controls, followed by qRT‑PCR validation and functional enrichment analysis.

**Results:**

Of 5,415 participants, 32 had RVO. The blood lead concentration in the RVO group was significantly higher than that in the control group (*p* < 0.001). In the fully adjusted model, higher blood lead levels were associated with increased odds of RVO (continuous model, *p* = 0.0111; top vs. bottom tertile: *p* = 0.0198).Cadmium and mercury showed no significant association. Multiple exposure analysis further confirmed that blood lead showed a stable risk contribution in the context of multiple exposure. RNA sequencing identified 166 common differentially expressed genes; PRICKLE4 and PLCXD1 expression changes were validated. Enrichment analysis revealed Wnt signaling pathway involvement, with PRICKLE4 linked to Wnt and PLCXD1 to phosphoric diester hydrolase activity.

**Discussion:**

These findings suggest lead may promote RVO by disrupting Wnt signaling and phosphodiesterase‑related pathways in endothelial cells, offering novel mechanistic insights.

## Graphical abstract

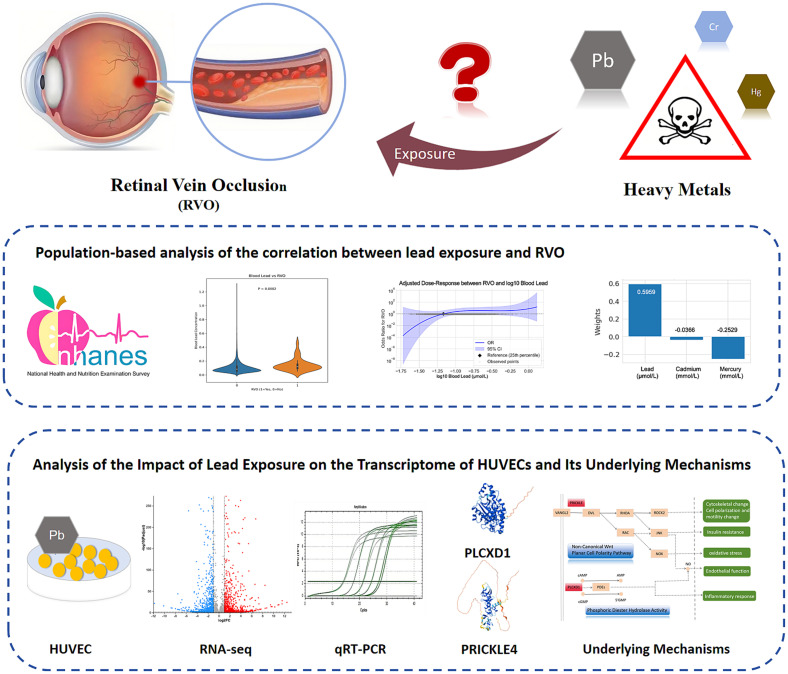

## 1 Introduction

Retinal vein occlusion (RVO) is one of the most common retinal vascular diseases and may lead to severe vision impairment or even blindness (1, 2). RVO often leads to complications such as sudden loss of monocular vision, macular edema, and retinal neovascularization, which seriously affect the patient’s quality of life. RVO is usually divided into central type (CRVO) and branch type (BRVO) according to the site of occlusion. Its etiology is complex and is generally believed to be closely related to hemodynamic changes, vascular wall damage, and hypercoagulable state (1, 2). However, the exact pathogenesis of RVO is still not fully understood. Some patients may develop the disease even without typical risk factors (such as hypertension, diabetes, and hyperlipidemia), suggesting that there may be other hidden environmental factors (2–4).

In recent years, with the acceleration of industrialization, environmental heavy metal pollution has received increasing attention due to its potential risk for human health. Heavy metals such as lead, mercury, cadmium, and arsenic can enter the human body through drinking water, air, food, etc., and have been proven to have multi-system toxicity, especially potential hazards to the cardiovascular system and nervous system (5, 6). It has been reported that heavy metals contribute to oxidative stress, endothelial dysfunction, and lipid metabolism disorders by exacerbating reactive oxygen species generation and inducing inflammation, thereby increasing the risks of hypertension, arrhythmias, and atherosclerosis (6–9). Notably, retinal vascular disorders are also closely associated with these pathogenic mechanisms (10–13). Therefore, heavy metal exposure may affect the stability of the retinal vascular system through the above shared mechanisms, thereby increasing the risk of RVO. However, direct epidemiological data on the relationship between heavy metal exposure and RVO are currently lacking.

Furthermore, previous studies have indicated that many lifestyle factors (e.g., body weight, smoking status) (14, 15) and underlying medical conditions (hypertension, hyperlipidemia) (14, 16) are also important risk factors for RVO. Therefore, in the statistical analysis of this study, in addition to focusing on the association between heavy metal exposure and RVO, we systematically evaluated the effects of traditional risk factors such as age, sex, body mass index (BMI), smoking status, hypertension, hyperlipidemia, and diabetes on RVO to control for potential confounding bias.

The National Health and Nutrition Examination Survey (NHANES) database is a large-scale, representative national population health survey project led by the Centers for Disease Control and Prevention (CDC). It covers a wealth of environmental exposure, laboratory testing and disease diagnosis information, and has been widely used to explore the potential link between the environment and chronic diseases (17, 18). Although existing studies have utilized NHANES data to explore the associations between heavy metals and cardiovascular diseases, kidney diseases, and other conditions, their relationship with retinal vascular diseases, particularly RVO, has not yet been systematically analyzed.

Therefore, this study utilized fundus examination and blood heavy metal testing data from individuals aged 40 and above in the NHANES database (2005–2008). A multivariate logistic regression model was employed to systematically evaluate the association between blood levels of lead, cadmium, mercury, and the risk of RVO. Additionally, a multi-pollutant model was applied to analyze their independent contributions in the context of mixed exposures. Furthermore, to explore the underlying mechanisms, we conducted *in vitro* experiments involving transcriptome sequencing of human umbilical vein endothelial cells (HUVECs) exposed to lead, followed by bioinformatics analysis and experimental validation. This approach aimed to identify key differentially expressed genes and analyze the signaling pathways potentially involved. The study seeks to provide new evidence from both epidemiological and molecular perspectives, thereby deepening the understanding of the environmental etiology of RVO.

## 2 Materials and methods

### 2.1 Data source and study population selection

The population-based cross-sectional analysis in this study is based on the NHANES project in the United States. It uses a population study and selects data from two cycles, 2005–2006 and 2007–2008. NHANES is led by the CDC in the United States. It uses a multi-stage stratified probability sampling method to represent the health and nutritional status of the non-institutionalized population in the United States. It contains detailed questionnaire information, clinical examinations, laboratory tests, and environmental exposure data. All participants signed a written informed consent before participating in the NHANES survey. This study initially included all respondents aged ≥40 years in the above two cycles to meet the high-risk age group of RVO epidemiological characteristics. Subsequently, RVO information was screened based on fundus imaging results, and individuals without RVO diagnosis records or blood heavy metal test results were further excluded.

### 2.2 Definition and diagnosis of retinal vein occlusion (RVO) in NHANES

During the 2005–2008 NHANES cycle, fundus photography was performed on respondents aged 40 years and older. Images were interpreted by professional assessors according to a standardized interpretation process, and changes related to retinal vein occlusion were recorded. The RVO outcomes were systematically classified using the following four coded variables: OPDDBVO (branch retinal vein occlusion in the right eye), OPDDCVO (central retinal vein occlusion in the right eye), OPDSBVO (branch retinal vein occlusion in the left eye), and OPDSCVO (central retinal vein occlusion in the left eye).

In this study, referring to previous literature and the official description of NHANES, any value of ≥2 in the above four variables was used as the basis for diagnosing RVO. That is, if any type of RVO (central or branch type) was confirmed in both eyes, they could be included in the RVO group.

In some analyses, RVO was further divided into BRVO and CRVO based on the variable values. Patients with both central and branch changes were no longer counted and classified as CRVO.

### 2.3 Determination of blood heavy metal level

This study selected blood and urine heavy metal test data provided in the NHANES database from 2005 to 2008 to evaluate individual heavy metal exposure levels. All tests were performed by certified laboratories designated by the National Center for Health Statistics (NCHS) of the United States using standardized methods and underwent strict quality control procedures. According to the description of the NHANES laboratory results section, blood samples were collected and tested using inductively coupled plasma mass spectrometry (ICP-MS) technology. The blood heavy metals included in this study include: Lead (Pb) (μmol/L), Cadmium (Cd) (nmol/L), and Mercury (Hg) (nmol/L). To reduce the influence of skewed heavy metal concentration distribution, a logarithmic 10 transformation is further carried when analyzing the effect of linear heavy metal concentration in RVO risk by logistic regression.

### 2.4 Selection and definition of covariates

To control potential confounding factors, this study included the following covariates in the multivariate analysis: age, sex, body mass index (BMI), smoking status, hypertension, diabetes, and hyperlipidemia. The definitions of each variable are as follows:

#### 2.4.1 Smoking status

Smoking status is categorized into three groups based on smoking queries (SMQ) from the questionnaire survey: Non-smokers: Individuals who have smoked less than 100 cigarettes in their lifetime (SMQ020 = 2); Ex-smokers: Those who had smoked ≥100 cigarettes (SMQ020 = 1) and were currently non-smokers (SMQ040 = 3); Current smokers: Including daily smokers (SMQ020 = 1 & SMQ040 = 1) and occasional smokers (SMQ040 = 2).

#### 2.4.2 Hypertension

Hypertension is determined by combining blood pressure measurement, questionnaire survey and medication use records. The specific definition is as follows: Those who meet any of the following conditions are considered to have hypertension: (1) Any systolic blood pressure (SBP, variables BPXSY1-3) ≥ 140 mmHg, or any diastolic blood pressure (DBP, variables BPXDI1-3) ≥ 90 mmHg in the three measurements; (2) Self-reported that a doctor has diagnosed hypertension (BPQ020 = 1); (3) Self-reported that they are taking antihypertensive drugs (BPQ040A = 1 or BPQ050 = 1), or antihypertensive drugs are included in the medication record.

#### 2.4.3 Diabetes

The definition of diabetes refers to the algorithm proposed by Varma et al. (19), combined with questionnaires, self-reporting, laboratory tests and drug use. Those who meet any of the following conditions are considered to have diabetes:

(1) Self-reported that they have been diagnosed with diabetes by a doctor;
(2) Glycated hemoglobin (HbA1c) value ≥ 6.5%;
(3) Self-reported that they are currently using insulin or oral antidiabetic drugs;
(4) Antidiabetic drugs are listed in the drug list.

#### 2.4.4 Hyperlipidemia

Hyperlipidemia is determined based on questionnaire responses and laboratory test results. Patients who met any of the following conditions were defined as having hyperlipidemia:

(1)Self-reported hyperlipidemia in the questionnaire (BPQ080 = 1);
(2) Laboratory test results showed LDL cholesterol (LBDLDL) ≥ 160 mg/dL, or total cholesterol (LBXTC) ≥ 240 mg/dL, or triglycerides (LBXTR) ≥ 200 mg/dL;
(3) Self-reported taking lipid-lowering drugs (BPQ090D = 1);
(4) The medication record contained lipid-lowering drugs.

### 2.5 Statistical analyses

All statistical analyses were performed in Jupyter Notebook using Python (version 3.12.2). First, the variables were standardized according to the NHANES data dictionary, and subjects aged ≥ 40 years were screened to construct a basic characteristic table of RVO patients and non-RVO controls. Continuous variables were expressed as medians (quartiles), and the Mann–Whitney U test was used for inter-group comparisons; categorical variables were expressed as frequencies (percentages), and the chi-square test was used for inter-group comparisons.

In the main effect analysis, to evaluate the association between the concentration of lead, cadmium, and mercury in the blood and RVO, both univariate and multivariate logistic regression model was constructed. The occurrence of RVO was taken as the dependent variable (0 = no, 1 = yes), and the heavy metal concentration was normalized by log10 conversion and was included in the model as a continuous variable. The confounding factors such as age, gender, BMI, smoking status, hypertension, diabetes mellitus (DM, prediabetes was classified into the normal group) and hyperlipidemia (HC) were adjusted, and the odds ratio (OR) and its 95% confidence interval were calculated.

In addition, to further explore the dose-effect relationship between heavy metal exposure and RVO risk, each heavy metal concentration was grouped by tertiles, and the lowest tertile (T1) was used as the reference. The highest tertile (T3) was subjected to classified logistic regression analysis to evaluate the risk increase of the high-exposure population.

To explore the possible nonlinear association between lead concentrations in blood and RVO, the restricted cubic spline (RCS) regression model based on the logistic regression framework was used to construct the dose–response relationship curve. The concentration of heavy metals was normalized into log 10 value. The RCS model was constructed using the patsy and stats models libraries in Python. An RCS curve was established for lead, and the reference value was set at the 25th percentile of the corresponding concentration. The model output included the OR of blood lead concentration to the risk of RVO and its 95% confidence interval (95% CI). The models were adjusted for age, body mass index (BMI), and sex (male vs. female). Considering the wide distribution range of lead concentrations, the horizontal axis used a logarithmic scale to improve the visualization and interpretability of the results.

Meanwhile, multiple exposure statistical models were used to comprehensively consider multiple heavy metal concentration variables to evaluate the potential synergistic effects and independent contributions of their combined exposure to RVO, thereby identifying key exposure factors. The weighted quantile sum (WQS) model was used to evaluate the joint association between heavy metal concentrations and the risk of RVO. The analysis included blood lead, cadmium, and mercury. The dataset was then divided into a training set (80%) and a test set (20%). In the implementation of the WQS model, a bootstrap method with 1,000 resamplings was used. For each resampling, the quantiles of heavy metal concentrations were calculated. Logistic regression models were fitted to these quantile-transformed data to estimate the coefficients of each heavy metal. The weights of each heavy metal were obtained by normalizing the coefficients of all bootstrap resamples. On the test set, the dot product of the quantile-transformed heavy metal concentrations and the obtained weights was calculated as the WQS score. Subsequently, a logistic regression model was established to evaluate the relationship between the WQS score and RVO, and the regression coefficients and their corresponding *p* values were calculated.

In terms of data visualization, the distribution differences of heavy metals in different groups of people were displayed by violin plots; the dose–response relationship was displayed by natural spline curves. All statistical tests were two-sided tests, and *p* values < 0.05 were considered statistically significant.

### 2.6 Cell culture and treatment

HUVECs were kindly provided by the Institute of Materia Medica, Chinese Academy of Medical Sciences and Peking Union Medical College. The cells were cultured in Dulbecco’s Modified Eagle Medium (DMEM; Procell, Wuhan, China) supplemented with 10% fetal bovine serum (FBS; Procell, Wuhan, China) and maintained in a 37 °C, 5% CO₂constant temperature and humidity incubator. When the cells reached 80% confluence, they were treated with two concentrations (10 μM and 30 μM) of lead nitrate (Aladdin, Shanghai, China) for 48 h.

### 2.7 RNA sequencing

The processed cells were collected and lysed using TRIzol (Invitrogen, USA) to extract total RNA, followed by assessment of RNA purity and integrity. Sequencing libraries were constructed according to the manufacturer’s instructions using the SMART-Seq_V4 Ultra Low Input RNA Kit for Sequencing (Clontech, USA). Sequencing was performed on the NovaSeq X plus platform (Illumina, USA). The expression levels of genes and transcripts were quantified using RSEM (http://deweylab.github.io/RSEM/), and differential expression analysis was conducted using the DEGseq 1.26.0 package (https://www.rdocumentation.org/packages/DEGseq/versions/).

### 2.8 Gene functional enrichment analysis

In mechanism analysis, Kyoto Encyclopedia of Genes and Genomes (KEGG) Pathway and Gene Ontology (GO) enrichment analyses are essential for functional annotation, helping to interpret the biological functions, molecular mechanisms, and regulatory networks of gene/protein sets.

KEGG identifies significantly enriched metabolic, signaling, and disease-related pathways, revealing the regulatory roles of gene sets in specific biological processes. GO is a systematic classification of gene functions, covering three levels: Molecular Function (MF), Cellular Component (CC), and Biological Process (BP), which, respectively, represent the molecular-level activities of genes, the subcellular structure or location of genes, and the biological events in which genes participate.

### 2.9 Quantitative real-time PCR analysis

Total RNA was reverse-transcribed into cDNA using the HiFiScript gDNA Removal RT MasterMix (Cwbio, Taizhou, China). qRT-PCR based on the Magic SYBR Mixture (Cwbio, Taizhou, China) was performed on a PCR platform (Bio-Rad, California, USA). The primer (Tsingke, Beijing, China) sequences are listed in Supplementary Table 1. The qRT-PCR results, with relative gene expression fold changes calculated by the 2^−ΔΔCt method normalized to GAPDH, were statistically analyzed using Prism 10.1.2 (GraphPad Software, Boston, USA). All data are expressed as mean ± SEM. Differences between two groups were assessed using the t-test, and *p* value of < 0.05 (*), < 0.01 (**), or < 0.001 (***) was considered statistically significant.

## 3 Results

### 3.1 Baseline characteristics of included participants

**Figure 1:**
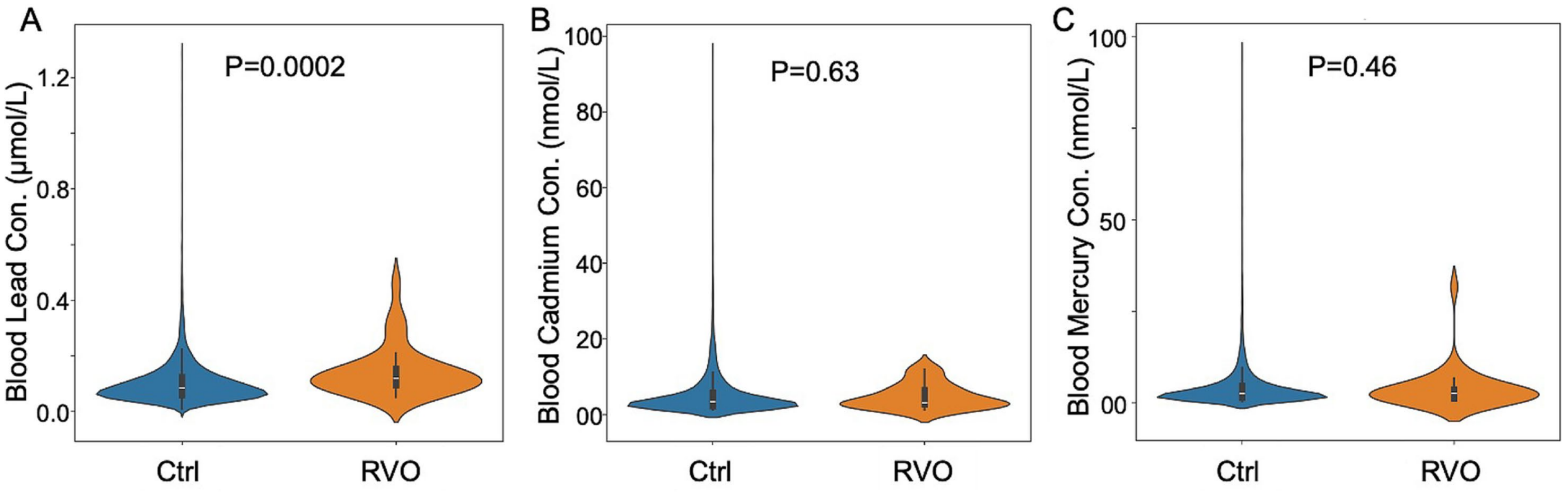
Distribution of blood heavy metal concentrations in participants with and without retinal vein occlusion (RVO). **(a)** Blood lead levels (μmol/L), **(b)** blood cadmium levels (nmol/L), and **(c)** blood mercury levels (nmol/L) were compared between RVO cases and controls using violin plots. Each violin plot displays the full distribution, median (white line), and interquartile range (black box). Statistical significance was assessed using the Mann–Whitney U test.

To investigate the association between blood heavy metal level and RVO, we used a public available NHANES survey data from 2005/2006 and 2007/2008 cycles. We included a total of 7,081 person aged more than 40. Subsequently, we excluded 1,500 participants due to the missing information in RVO documentation (5,581 participants left). For blood heavy metal analysis (lead, cadmium and mercury), further 166 participants were then excluded due to the lack of blood heavy metal. Eventually, a total of 5,415 individuals were included in this study. Baseline characteristics of included participants were shown in Table 1. Subjects with RVO were older than control (*p*<0.0001). Blood lead concentration was significantly higher in patients with RVO (*p*=0.0002, MW-U test). Blood cadmium and mercury showed no significant difference between RVO and control individuals (Figure 1).

**Table 1:** Baseline characteristics of included participants.

Variable	RVO Group (*n* = 32)	Non - RVO Group (*n* = 5,383)	*p* value
Male (number (%))	19 (59.38%)	2,676 (49.71%)	0.36
Age (years)	68.84 ± 12.09	59.40 ± 12.38	<0.001^*^
BMI (kg/m^2^)	27.73 ± 5.33	29.27 ± 6.46	0.17
Smoking status			0.831
Never	14	2,541	
Current	6	1,093	
Former	12	1746	
Hypertension			0.087
Yes	24	3,149	
No	8	2,234	
Diabetes mellitus			0.60
Yes	8	1,054	
No	24	4,249	
Borderline	0	80	
Hyperlipidemia			1.00
Yes	19	3,117	
No	13	2,266	
Blood Lead (μmol/L)	0.14±0.09	0.10±0.08	0.0002^*^
Blood Cadmium (nmol/L)	4.83±3.32	5.17±5.61	0.63
Blood Mercury (nmol/L)	7.50±11.24	7.99±10.73	0.46

Differences in continuous variables between the RVO and non-RVO groups were assessed using the Mann–Whitney U test due to non-normal distributions. Categorical variables were compared using the Chi-square test.

*indicates a statistically significant difference (*p* value < 0.05).

### 3.2 Single metal exposures and RVO risk

In univariate logistic regression analysis, log10-transformed blood lead was associated with a significantly increased risk of RVO: the OR for RVO was 2.705 (95% CI: 1.595–4.587, *p*=0.0002); comparing the highest tertile of blood lead with the lowest tertile, the OR was 9.15 (95% CI: 2.12–39.47, *p*= 0.003). After adjusting for confounding factors such as age, sex, BMI, smoking status, hypertension, diabetes, and hyperlipidemia, the relationship between blood lead level and RVO remained statistically significant, with a continuous variable OR of 2.189 (95% CI: 1.196–4.007, *p*=0.0111) and an tertile comparison OR of 6.16 (95% CI: 1.34–28.39, *p*=0.0198) (Table 2).

**Table 2:** Associations of single blood heavy metals with RVO.

Exposure (model type)	Log 10 Continuous OR (95% CI)	*p* value	Top tertile vs. bottom tertile OR (95% CI)	*p* value
Blood lead				
Univariable	2.705 (1.595, 4.587)	0.0002^*^	9.15 (2.12, 39.47)	0.003^*^
Multivariable	2.189 (1.196, 4.007)	0.0111^*^	6.16 (1.34, 28.39)	0.0198^*^
Blood cadmium				
Univariable	1.058 (0.669, 1.676)	0.81	1.87 (0.75, 4.70)	0.18
Multivariable	0.877 (0.449, 1.713)	0.70	1.33 (0.44, 4.07)	0.61
Blood mercury				
Univariable	0.855 (0.585, 1.251)	0.42	0.63 (0.24, 1.64)	0.34
Multivariable	0.922 (0.628, 1.354)	0.68	0.77 (0.29, 2.04)	0.60

Risk of blood lead, cadmium, mercury on RVO were calculated with logistic regression model. Heavy metals were treated as both continuous and categorical variables, respectively.

For multivariable models, covariables of age, gender, BMI, smoking status, hypertension, DM, hyperlipidemia were included for adjustment. OR, odds ratio. CI, confident intervals.

*indicates a statistically significant difference (*p* value < 0.05).

In contrast, blood cadmium and blood mercury were not significantly associated with the risk of RVO. The adjusted OR for the continuous variable of log10-transformed blood cadmium was 0.877 (95% CI: 0.449–1.713, *p*=0.70), and the OR for tertile comparison was 1.33 (95% CI: 0.44–4.07, *p*=0.61); the adjusted OR for blood mercury was 0.922 (95% CI: 0.628–1.354, *p*=0.99), and the OR for tertile comparison was 0.77 (95% CI: 0.29–2.04, *p*=0.60) (Table 2). These results suggest that elevated blood lead levels may be an independent risk factor for RVO, while there is no significant association between blood cadmium and blood mercury and RVO.

**Figure 2:**
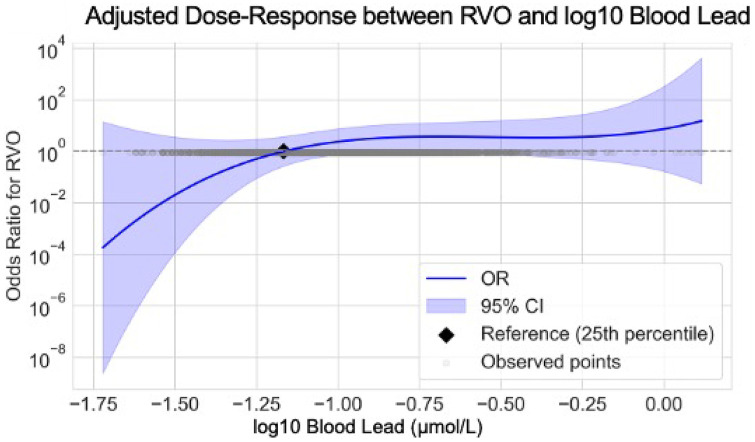
Adjusted odds ratios (ORs) and 95% confidence intervals (CIs) for RVO across the range of blood lead concentrations. Restricted cubic spline logistic regression models were used to estimate the associations between blood concentrations of lead (μmol/L) and the risk of RVO. The blue lines represent the adjusted ORs, and the shaded areas indicate the 95% confidence intervals. The reference point (black dot) was set at the 25th percentile of lead concentration distribution. Age, sex, and BMI were adjusted in this model.

To explore the possible nonlinear relationship between blood lead and RVO risk, we constructed a restricted cubic spline (RCS) model based on log₁₀ concentration of blood lead. Although the dose–response relationship was not statistically significant, the risk curve showed that the risk of RVO increased with increasing blood lead concentration, which was consistent with the results of logistic regression (Figure 2). In the supplementary analysis, we further used the RCS model to evaluate the nonlinear dose–response relationship between blood cadmium and blood mercury levels and the risk of RVO (Supplementary Figures S1A,B). The dose–response curves showed that there was no obvious increasing trend with the risk of RVO in the entire range of log10 transformed blood cadmium or mercury concentrations.

### 3.3 Multi-metal exposures and RVO risk

The regression coefficient of the relationship between the WQS score and RVO was 0.4866 with a *p* value of 0.944. This indicated, based on the current sample and statistical analysis, there was no statistically significant association between the combined effect of heavy metals and RVO.

**Figure 3:**
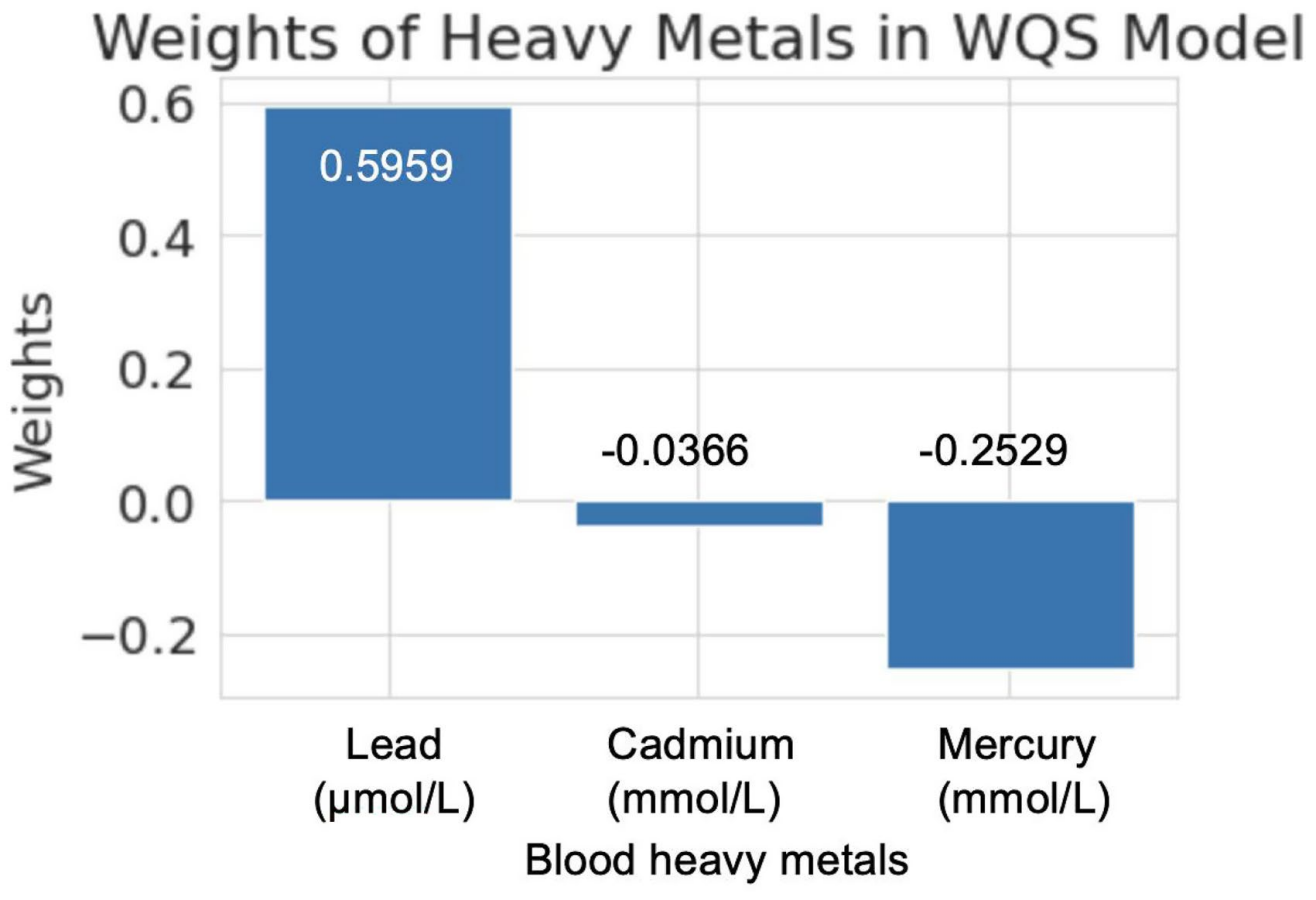
Weights of individual heavy metals in the WQS regression model evaluating the combined effect of blood lead, cadmium, and mercury on the risk of RVO.

The weights of the three heavy metals in the WQS model were as follows (Figure 3): lead had a weight of 0.5959, indicating a positive association with RVO risk, suggesting that an increase in its concentration might contribute to a higher risk of RVO. Blood cadmium had a weight of - 0.0366, showing a weak negative tendency, meaning that an increase in its concentration might slightly reduce the risk of RVO, albeit marginally. Blood mercury had a weight of - 0.2529, also indicating a negative impact on RVO risk, but with a lesser influence compared to the positive effect of blood lead.

### 3.4 Transcriptomic changes in HUVECs induced by lead nitrate

**Figure 4:**
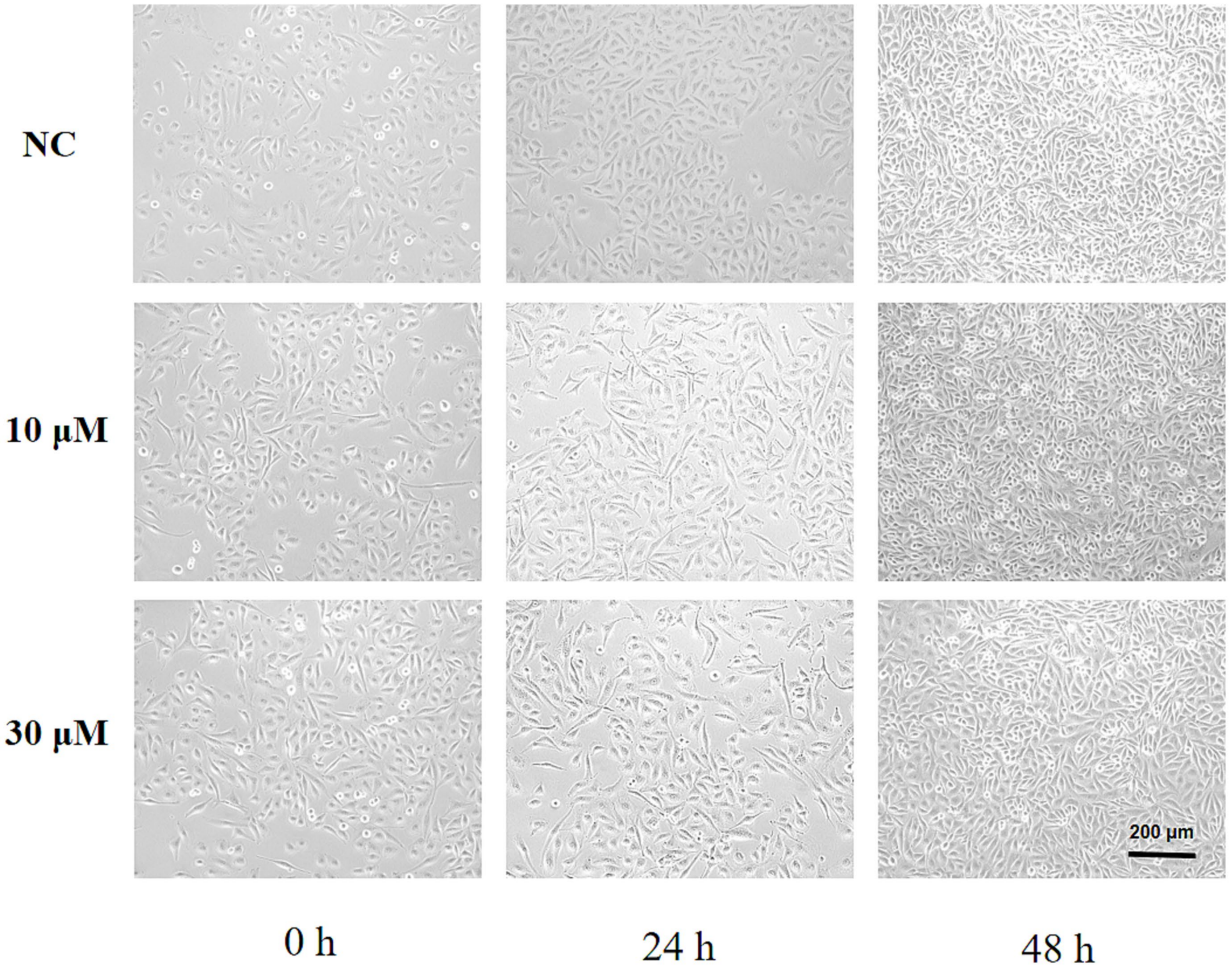
Photomicrographs of HUVEC cell lines exposed to two concentrations (10 μM and 30 μM) of lead nitrate for 24 and 48 h.

HUVEC cells were treated with 10 μM and 30 μM lead nitrate. The microscopic pictures of the two lead nitrate-treated groups and the normal control (NC) cells at 0 h, 24 h, and 48 h are shown in Figure 4.

**Figure 5:**
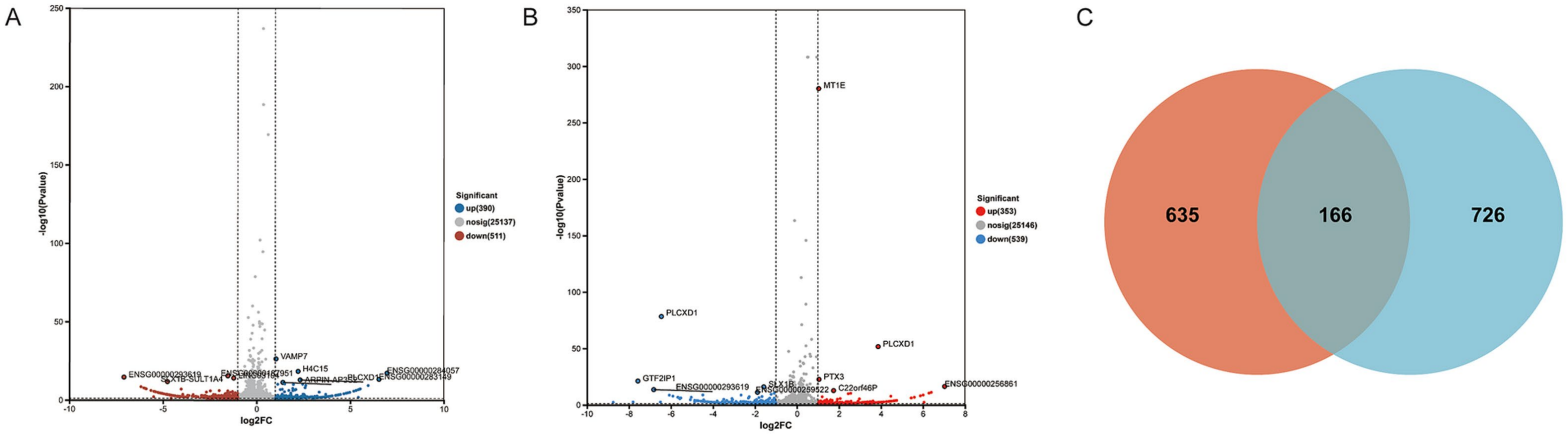
Transcriptomic changes in HUVECs after lead nitrate treatment. Panel **(a,b)** show the DEGs for the 10 μM and 30 μM lead nitrate treatments, respectively, compared to the NC group. **(c)** Illustrates the overlap of DEGs between the two treatment groups; the red area represents DEGs unique to the 10 μM vs. NC comparison, the green area represents DEGs unique to the 30 μM vs. NC comparison, and the central overlapping region indicates the shared DEGs.

RNA sequencing was performed to detect gene expression in samples treated with 10 μM and 30 μM lead nitrate compared to NC samples. Differentially expressed genes (DEGs) were statistically analyzed, as shown in the volcano plots (Figures 5a,b). Compared with the NC samples, the 10 μM lead nitrate-treated group showed 390 upregulated genes and 511 downregulated genes. Among these, vesicle associated membrane protein 7 (VAMP7) was the most significantly regulated gene. In comparison with the NC samples, the 30 μM lead nitrate-treated group exhibited 353 upregulated genes and 539 downregulated genes. The most significantly regulated genes in this group were metallothionein 1E (MT1E) and phosphatidylinositol specific phospholipase C X domain containing 1 (PLCXD1).

The intersection of DEGs from the 10 μM lead nitrate-treated vs. NC comparison and the 30 μM lead nitrate-treated vs. NC comparison was analyzed, and a Venn diagram was constructed (Figure 5c), revealing 166 overlapping genes. Detailed information on these 166 genes is provided in Supplementary Table 2.

### 3.5 qRT-PCR validation of candidate DEGs

From the aforementioned 166 genes, we applied the following criteria for further screening: genes showing dose-dependent normalized counts across the untreated, 10 μM lead nitrate-treated, and 30 μM lead nitrate-treated samples; genes with normalized counts greater than 1 in at least one of the three samples; and genes documented in the literature as being associated with the “eye.” Six genes meeting these criteria were ultimately selected for further validation via qRT-PCR. Detailed information on these genes is provided in Table 3.

**Table 3:** Detailed information of six genes validated by qRT-PCR.

Gene name	Gene description	Normalized counts		
		HUVEC_NC	HUVEC_10μM	HUVEC_30μM
SAXO2	stabilizer of axonemal microtubules 2 [Source: HGNC Symbol; Acc: HGNC:33727]	1.36	0.22	0.04
HSD11B2	hydroxysteroid 11-beta dehydrogenase 2 [Source: HGNC Symbol; Acc: HGNC:5209]	2.51	1.36	0.55
PRICKLE4	prickle planar cell polarity protein 4 [Source: HGNC Symbol; Acc: HGNC:16805]	2.52	1.04	0.97
PLCXD1	phosphatidylinositol specific phospholipase C X domain containing 1 [Source: HGNC Symbol; Acc: HGNC:23148]	4.83	2.18	1.12
IKBKGP1	inhibitor of nuclear factor kappa B kinase subunit gamma pseudogene 1 [Source: HGNC Symbol; Acc: HGNC:24455]	1.95	4.8	5.41
GP1BB	glycoprotein Ib platelet subunit beta [Source: HGNC Symbol; Acc: HGNC:4440]	1.17	2.86	4.81

**Figure 6:**
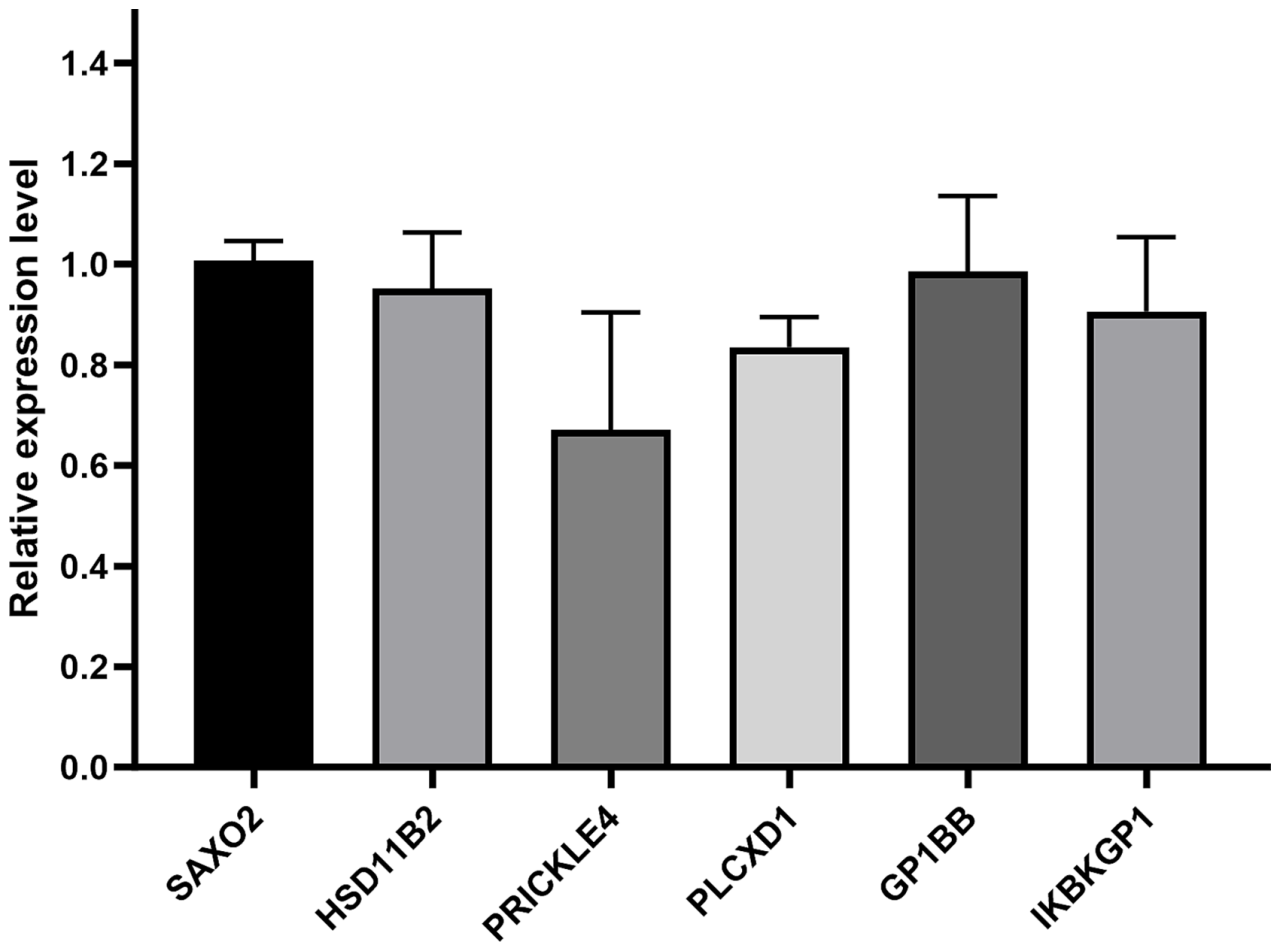
Comparison of the relative expression levels of six genes in HUVECs treated with or without lead nitrate.

The expression changes of the six candidate genes in the qRT-PCR results are shown in the bar graph in Figure 6. Among them, PRICKLE4 and PLCXD1 exhibited statistically significant differential expression, with *p* values of 3.07 × 10^−2^and 1.56 × 10^−3^, respectively. The other four genes did not show significant differences.

### 3.6 Functional enrichment analysis of key DEGs

To elucidate the biological functions and molecular mechanisms of lead-induced RVO, KEGG and GO analyses were performed on the 166 overlapping genes.

**Figure 7:**
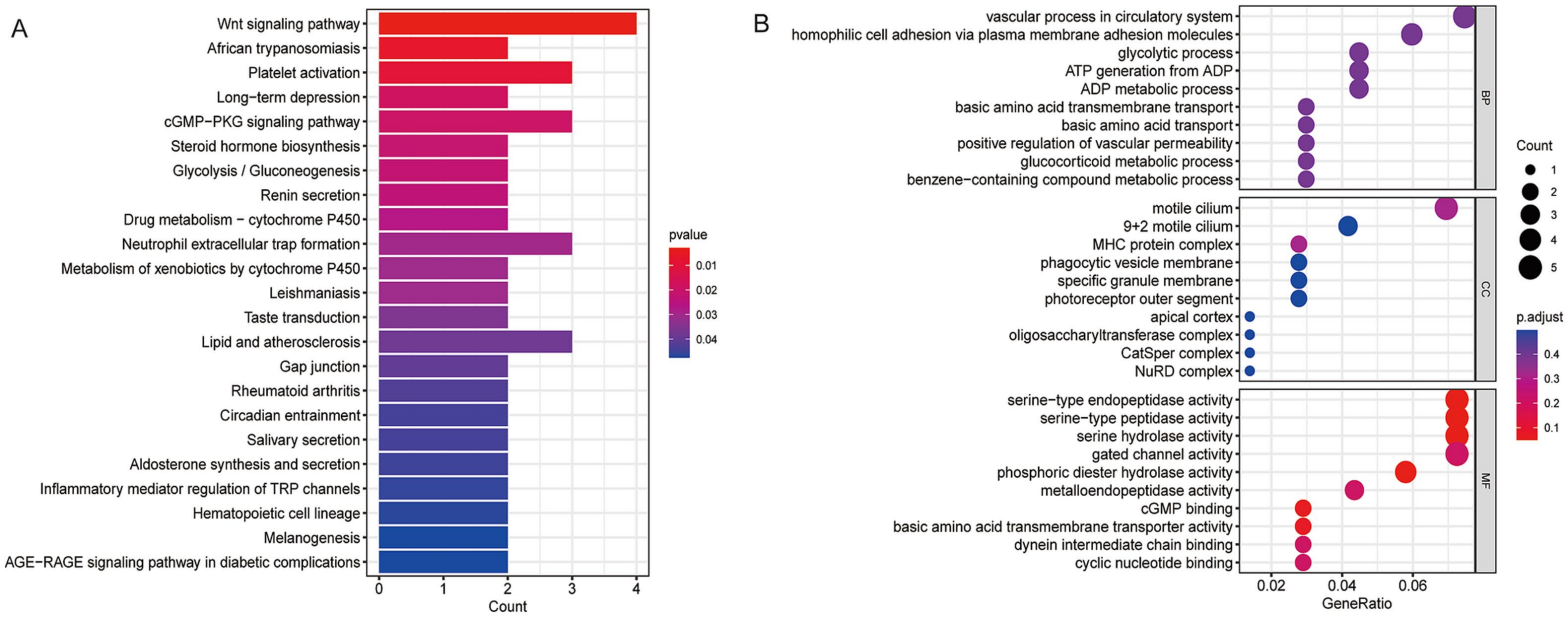
Mechanistic analysis of lead-induced RVO. **(a)** KEGG pathway analysis of potential target genes; **(b)** GO analysis showing the top 10 terms in BP, CC, and MF categories.

The KEGG pathway results (Figure 7a) indicate that the 166 genes were significantly enriched in 23 pathways (*p* value < 0.05). Detailed enrichment information is provided in Supplementary Table 3. The vertical axis represents pathway names, and the horizontal axis indicates the number of genes. The length of each bar corresponds to the number of overlapping genes enriched in a given pathway, longer bars represent a greater number of enriched genes. The color gradient (from blue to red) reflects decreasing *p* values, with redder colors indicating higher significance of pathway enrichment. Among these, the Wnt signaling pathway contained the largest number of genes (4 genes) and displayed the reddest color, indicating the most significant enrichment (*p* value = 0.0029). Notably, the validated gene PRICKLE4 was present and exclusively mapped to the Wnt signaling pathway.

Additionally, the GO analysis results (Figure 7b) show the top 10 ranked terms for BP, CC, and MF. Only four MF terms exhibited significant differences (*p*.adjust < 0.05), with detailed information listed in Supplementary Table 4. Among these four, phosphoric diester hydrolase activity was one of the MF terms with the highest significance (*p*.adjust = 0.0491). It is also noteworthy that the other validated gene, PLCXD1, was positively enriched in and exclusively associated with this GO term.

## 4 Discussion

As a common environmental toxin, lead has multiple effects such as vasoconstrictio (7, 20, 21), endothelial damage (7, 8, 22), and induction of oxidative stress (6, 9, 23). Studies have shown that it can promote the formation of atherosclerosis (24–26) and is associated with an increased risk of coronary heart disease and stroke (27, 28).

Based on the large population database of NHANES, this study systematically evaluated the relationship between blood heavy metals and RVO for the first time. The results showed that the blood lead level of patients with RVO was significantly higher than that of the control group. After adjusting for confounding factors such as age, gender, BMI, smoking status, hypertension, hyperlipidemia and diabetes, the results of logistic regression analysis showed that elevated blood lead concentration was still significantly associated with the occurrence of RVO, suggesting that blood lead may be an independent risk factor for RVO. This study, through combined RNA-seq and qRT-PCR analysis, successfully identified and validated PRICKLE4 and PLCXD1 as two key response genes in HUVECs exposed to lead nitrate. Functional enrichment analysis further revealed that these genes are closely associated with the “Wnt signaling pathway” and “phosphoric diester hydrolase activity” respectively.

From a mechanistic perspective, lead is a widely present environmental pollutant with a variety of known toxicological effects, especially in the cardiovascular system (6, 28). Previous studies have shown that lead can participate in the occurrence and development of a variety of vascular diseases, such as stroke (27), and atherosclerosis (24, 26), by inducing oxidative stress (9, 23), damaging vascular endothelium (22), activating inflammatory pathways (8), and promoting vasoconstriction and thrombosis. As a venous obstructive fundus disease, the pathogenesis of RVO also involves venous blood flow stasis, vascular wall damage and hypercoagulability, which can theoretically be affected by the above-mentioned lead toxicity mechanism. The results of this study provide epidemiological evidence for this hypothesis.

**Figure 8:**
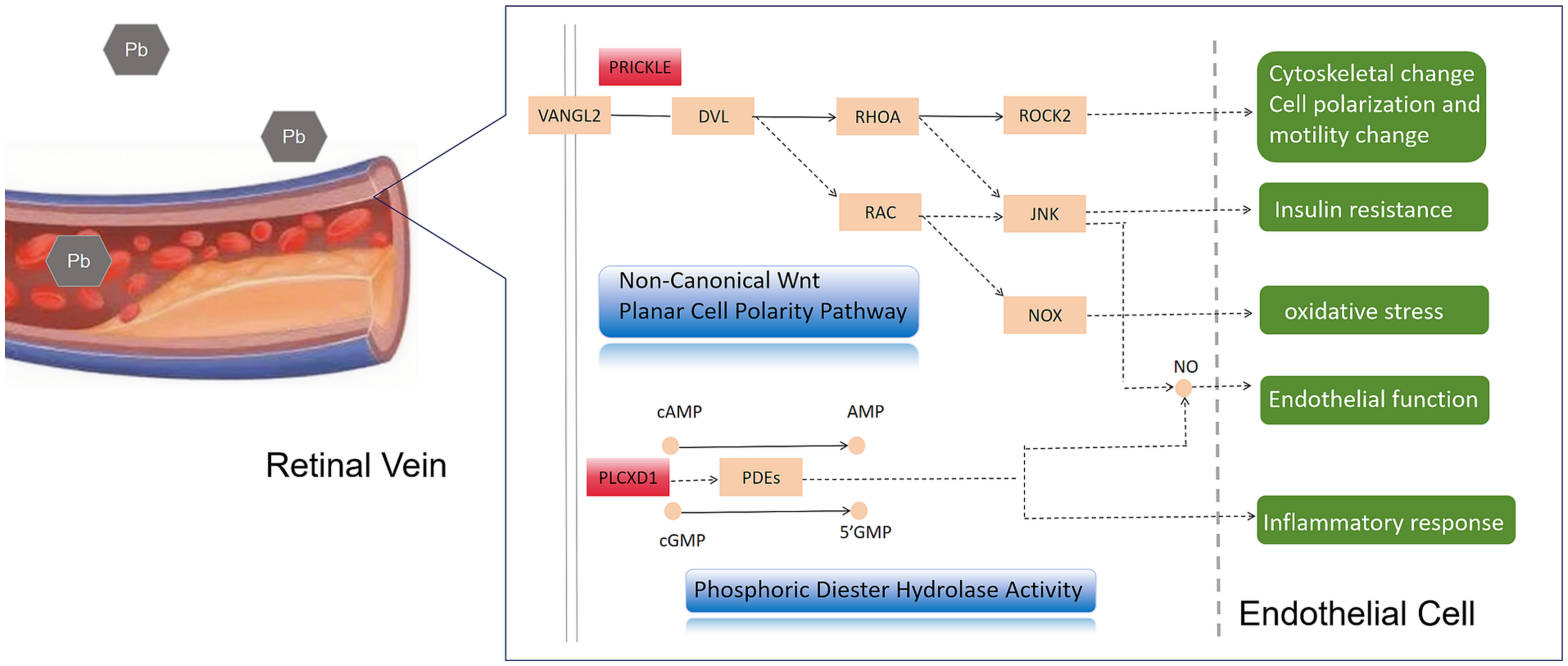
Potential mechanisms of lead exposure leading to RVO.

Our results indicate that PRICKLE4 is significantly enriched in the “Wnt signaling pathway.” PRICKLE proteins serve as core components of the non-canonical Wnt planar cell polarity pathway, which regulates epithelial polarity and cell migration (29). This pathway (Figure 8) can modulate cytoskeletal dynamics and cell motility by activating small GTPases such as RAC1 and RHOA (30). It also plays a crucial role in retinal vascular development and maintenance, being essential for the formation of the anterior neural plate, lens, and retinal ganglion cell (RGC) axon outgrowth (31, 32). Notably, the Wnt signaling pathway has been implicated in various vascular pathological processes, including oxidative stress, insulin resistance, and endothelial dysfunction (30). Specifically, WNT-mediated activation of RAC1 can influence oxidative stress by stimulating NADPH oxidase (NOX) activity (33). Wnt signaling may also induce insulin resistance through JNK-dependent suppression of AKT and eNOS phosphorylation (34–36), and it can activate inflammatory factors such as NF-κB and tumor necrosis factor, indirectly affecting vascular function (37, 38). Furthermore, non-canonical Wnt-mediated JNK activation modulates AKT phosphorylation, eNOS activity, nitric oxide (NO) bioavailability, and ultimately endothelial functions such as vasodilation and blood flow regulation (33, 39, 40). Therefore, lead nitrate may upregulate PRICKLE4, thereby disrupting non-canonical Wnt signaling, impairing endothelial cell homeostasis, and contributing to the pathological progression of RVO.

GO analysis indicates that the molecular function of PLCXD1 is significantly enriched in “phosphoric diester hydrolase activity.” Phosphodiesterases (PDEs) are a superfamily of enzymes that hydrolyze cyclic nucleotides (cAMP/cGMP) and are involved in regulating the metabolic balance of intracellular second messengers. Dysregulation of their activity is closely associated with various vascular diseases (41, 42). For example, inhibition of PDE4 can modulate regulatory T-cell function and exert anti-inflammatory effects (43). Meanwhile, PDE5 is highly expressed in smooth muscle and blood vessels, where it regulates cGMP levels and synergizes with NO signaling to modulate intracellular calcium homeostasis, thereby influencing vasodilation and optic nerve blood supply (44, 45). Clinical studies have shown that PDE5 inhibitors (such as sildenafil) exert certain effects on patients with non-arteritic anterior ischemic optic neuropathy and other ocular conditions (45). Therefore, altered expression of PLCXD1 may interfere with PDEs-related activity, disrupt cAMP/cGMP homeostasis, and subsequently affect vascular tone and local microcirculation. This could represent one of the mechanisms through which lead exposure contributes to retinal venous hemodynamic disturbances.

Although PRICKLE4 and PLCXD1 belong to distinct pathways, they may exert synergistic effects in regulating vascular function. For instance, both non-canonical Wnt signaling and PDE/cGMP signaling ultimately influence NO signaling and endothelial function. We speculate that under lead exposure, PRICKLE4-mediated disruption of cytoskeletal organization and cell polarity, combined with PLCXD1-related disturbances in second-messenger metabolism, collectively exacerbate endothelial dysfunction, abnormal vascular tone, and hemodynamic alterations, ultimately contributing to the onset and progression of RVO.

This study has several limitations that should be considered. First, this study is based on the NHANES database, in which the fundus examination methods are unable to assess or distinguish subtle changes such as subclinical alterations like paracentral acute middle maculopathy (PAMM) or retinal ischaemic perivascular lesions (RIPL). We acknowledge that even in the non-RVO group, these subclinical alterations may exist and could serve as predictive markers of vascular and systemic integrity. Second, because these findings are derived solely from cellular experiments, further validation through *in vivo* models is required. Third, although gene expression and bioinformatics analyses have been performed, the precise biological functions and regulatory networks of the identified genes remain to be elucidated. Thirdly, this study was limited by its retrospective design and the use of relatively old data, which may have introduced selection and information biases and may reduce the contemporaneous generalizability of the findings. To address the above limitations and advance subsequent research, future efforts can be pursued in the following directions: (1) initiating a prospective study utilizing higher-resolution fundus imaging techniques, such as optical coherence tomography angiography (OCTA), to explore the association between heavy metal exposure and these subclinical retinal microvascular alterations, which would facilitate earlier identification of high-risk populations; (2) modulating the expression of PRICKLE4 and PLCXD1 in HUVECs to assess their effects on lead-induced phenotypic changes; (3) employing western blotting and immunofluorescence assays to determine whether PRICKLE4 affects key Wnt signaling components, such as DVL and RAC1; (4) characterizing the enzymatic substrates of PLCXD1 and evaluating potential changes in its activity under pharmacological intervention; and (5) establishing an animal model of lead exposure to functionally validate the contribution of these genes to RVO pathogenesis *in vivo*.

## 5 Conclusion

In summary, this study is the first to identify PRICKLE4 and PLCXD1 as key responsive genes in vascular endothelial cells exposed to lead. These two genes may synergistically participate in lead-induced endothelial dysfunction and the pathological process of retinal vein occlusion through mechanisms related to the non-canonical Wnt signaling pathway and phosphoric diester hydrolase activity, respectively. This provides new experimental evidence and potential therapeutic targets for further elucidating the molecular mechanisms of lead-induced RVO.

## Data Availability

The datasets presented in this study can be found in online repositories. The names of the repository/repositories and accession number(s) can be found in the article/Supplementary material.

## Supplementary material

SUPPLEMENTARY FIGURE S1 Adjusted odds ratios (ORs) and 95% confidence intervals (CIs) for RVO across the range of blood cadmium **(A)** and blood mercury; **(B)** concentrations.
