# Supplementary figures and images for: Lead exposure increases the risk of retinal vein occlusion: a population-based analysis and investigation of PRICKLE4/PLCXD1-mediated endothelial cell mechanisms
Source: Front Public Health. 2026 May 12;14:1826278. doi: 10.3389/fpubh.2026.1826278 (PMC13201478; doi:10.3389/fpubh.2026.1826278)

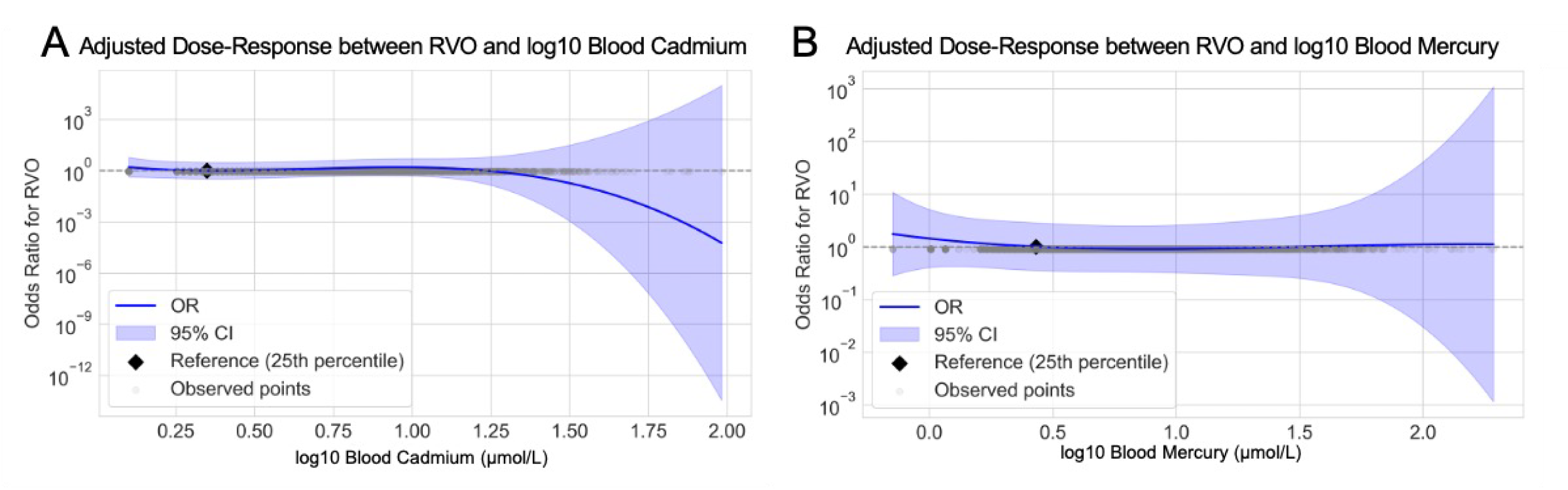

Supplement: SUPPLEMENTARY FIGURE S1 — Adjusted odds ratios (ORs) and 95% confidence intervals (CIs) for RVO across the range of blood cadmium (A) and blood mercury; (B) concentrations. [file Image_1.tif]
